# Probiotics: A Promising Role in Dental Health

**DOI:** 10.3390/dj5040026

**Published:** 2017-09-27

**Authors:** Sari A. Mahasneh, Adel M. Mahasneh

**Affiliations:** 1 School of Dental Medicine, The University of Manchester, Manchester, M13 9PL, UK; sari-mahasneh@hotmail.com; 2 Department of Biological Sciences, The University of Jordan, Amman 11942, Jordan

**Keywords:** probiotics, *Lactobacillus*, dental healthcare, periodontitis, caries, halitosis

## Abstract

Probiotics have a role in maintaining oral health through interaction with oral microbiome, thus contributing to healthy microbial equilibrium. The nature and composition of any individual microbiome impacts the general health, being a major contributor to oral health. The emergence of drug resistance and the side effects of available antimicrobials have restricted their use in an array of prophylactic options. Indeed, some new strategies to prevent oral diseases are based on manipulating oral microbiota, which is provided by probiotics. Currently, no sufficient substantial evidence exists to support the use of probiotics to prevent, treat or manage oral cavity diseases. At present, probiotic use did not cause adverse effects or increased risks of caries or periodontal diseases. This implicates no strong evidence against treatment using probiotics. In this review, we try to explore the use of probiotics in prevention, treatment and management of some oral cavity diseases and the possibilities of developing designer probiotics for the next generation of oral and throat complimentary healthcare.

## 1. Introduction

It is well recognized that the human microbiome including bacteria, fungi, and viruses is ten times the number of cells of our body [1,2]. For obvious reasons, great attention in healthcare focused on the gut microbiota until recently, when microbial populations of other body regions, especially of the oral cavity, became of concern [3,4].

In the oral cavity, a diverse population has been estimated to include more than 700–1000 bacterial species spread on the tongue, teeth, gum, inner cheeks, palate and tonsils. Streptococci form about 20% of these bacteria, in addition to viruses, fungi and some archaea. It is generally accepted that oral health is affected by residing bacteria as well as the individual’s age, health, nutritional status and lifestyle [5].

With the slow progress of isolating new antibiotics coupled with the increase of emerging resistant pathogenic bacteria, it has become imperative to try and enhance the use of living therapeutics. Probiotics form the cornerstone of such biotherapy. According to World Health Organization, the definition of probiotics refers to “live microorganisms which when administered in adequate amounts, confer benefits to the health of the host”. Probiotic effect on human health has been substantiated for many years [6]. Research results have confirmed the positive activity of probiotic lactic acid bacteria in prevention and treatment of antibiotic associated diarrhea rota virus infections and many gastrointestinal diseases [7]. It is also known that probiotic bacteria including lactobacilli and bifidobacteria are good colonizers of the gastrointestinal tract, vagina and oral cavity of humans [8], which broaden the prospective role of biotherapy. On the other hand, recent studies suggested a role of periodontopathic bacteria in enhancing systemic diseases including diabetes, respiratory and cardiovascular cases [9]. Probiotic preparations are increasingly used to confer good health substantiated with successful randomized clinical trials. Table 1 presents results of some randomized clinical trials on the use of probiotics in clinical applications. Table 1 shows the little attention given to the use of probiotics for a healthy oral cavity. However, in the last decade, more research has been carried out in this direction and it was extended to the oral cavity where probiotics are expected to play an important therapeutic and/or preventive role in the development of oral diseases.

## 2. Foreseen and Research Targeted Activities of Probiotics

The envisaged role of probiotic bacteria on human health is associated with remarks of Metchnikoff, a Nobel Prize winner, at the turn of the 20th century on longevity of peasants relying on fermented dairy products as a major diet component. It is now well established that some probiotic bacteria, mainly *Lactobacillus* and *Bifidobacterium* confer health benefits. This is substantiated through continued research and randomized clinical trials [5,19,20]. Probiotics benefits tend to be widened with the development of more accurate research methods to better understand microbe–host interactions [21]. Such interactions include, but are not limited to, modulation of the immune responses [22], strengthening means of evading pathogens [23], affecting the balance of the host microbiota [24] and the metabolism of microbiota at specific locations of the host body [25,26]. All of the above observations led researchers to revisit the selection criteria of probiotics with great emphasis on the ability: (1) to harness the immune response, whether it is specific or non-specific; (2) to produce antimicrobials including bacteriocins; (3) to compete successfully for binding site; and (4) to survive during oro-gastro intestinal passage, thus resisting host defense mechanisms with assured safety to the users. Considering the accumulating literature about the proven benefits of probiotics and their inhibitory effects on the growth of pathogenic microorganisms [27,28,29], researchers have extended their interest to include the oral cavity where probiotics may exhibit some therapeutic or preventive outcome on the incidence and progression of oral diseases. Consequently, regarding the oral environment, some studies on oral probiotic bacteria role on prevention and control of dental caries have been promising [30,31,32]. Indeed, it is recognized that most new strategies to deal with oral cavity diseases are based on manipulating microbial activities through use of beneficial probiotics inhibitory activities against pathogens including cariogenic and gingivitis causing microbiota [21,26,27]. In this review, we present information on the expected beneficial role of probiotic microorganisms in the oral cavity in the context of the wider scope of the new era of biotherapeutics or biotherapy. 

## 3. Possible Roles of Probiotics in the Oral Cavity 

Accumulating research results point to the following activities of probiotics in the human oral cavity:

(1) Antagonism with pathogens;
(2) Aggregation with oral bacteria; and
(3) Interaction with oral epithelium.

In Cases 1 and 2, it is expected that, through such processes, modulation of the oral–biofilm composition will take place [33,34,35,36]. This would result in reducing the pathogenicity and cariogenic potential of biofilm microorganisms [33,37,38,39] as well as reducing the potential pathogens burden in oral biofilm [39,40,41]. The final outcome will definitely present a clear path for caries, gingivitis and periodontal management [42,43,44,45,46].

**Figure 1:**
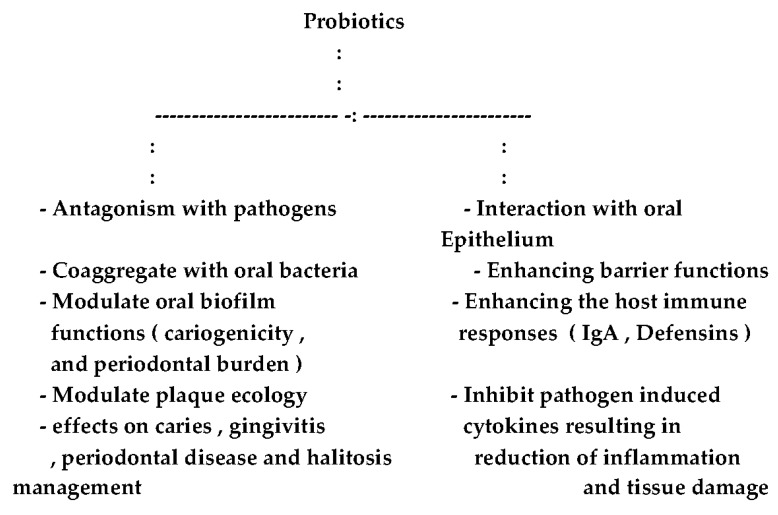
The envisaged probiotic roles in the oral cavity.

As for interaction with oral epithelium, research results point out the ability of the probiotic bacteria to strengthen the epithelial barrier function [16,47,48,49], in addition to modulating the innate and adaptive immune responses [50,51,52]. Figure 1 summarizes the probable combined effects of probiotics on oral health.

## 4. Oral Microbiota Characteristics 

The oral microbiota develops emergent characteristics that cannot be observed from studies of single species [43,44,46,48]. This microbiota, in regards to its structure and function, is highly organized and is considered similar to multicellular organism by some researchers [44]. Usually, it is recognized in a healthy setting that the numerous interactions would contribute to resilience and stability of an ecosystem against perturbations. Consequently, if certain pathogenesis parameters that vary among patients exceed threshold, competitiveness among bacteria will be altered leading to caries and periodontal diseases [48]. Understanding of these situations will without doubt lead to strategies of better oral health control and management [45].

The tremendous nutritional and physical interactions that develop among and within the species during infection are greatly affected by an array of host factors eliciting inflammatory responses. Many studies indicated a great biomass yield when more than one species are grown in co-culture [53]. This has been explained by species responding to the presence of each other through changes in the rate of certain gene expression. Other studies highlighted the interaction role of close physical associations to biofilm formation [54].

## 5. The Oral Cavity and Indigenous Probiotics

Considering all of the above, the question of the presence of probiotic bacteria within the indigenous oral microbiota has been partially answered. Recent studies mention no less than 1000 bacterial species residing or transient within the oral cavity [49,55]. These bacteria in the cavity are either planktonic or integrated into an oral biofilm of diverse oral surfaces or niches. In this context, research results recorded major physiological differences between bacteria of the planktonic state and those in biofilms [43,56,57]. Keigser et al. [55] reported the presence of more than 1000 species in the oral cavity in both planktonic and biofilm statuses. Saliva also contributed to the microbial diversity through its composition and easy flow and flow effect upon continuous detachment of bacterial cells from biofilm surfaces. The question of presence of probiotics indigenously in the mouth is still unclear; however, since *Lactobacillus* and *Bifidobacterium* form the majority of probiotics in general, it could be said that some lactobacilli in the mouth would exhibit beneficial effects [58]. Although information regarding indigenous probiotics in the oral cavity is very scarce [59], Koll-Klais et al. [60] reported that healthy oral cavity was populated with *Lactobacillus gasseri* and *Lactbacillus fermentum*, whereas periodontitis patients were free of these two species but populated with *Lactobacillus plantarum*. Ample clinical studies presented results indicating the positive effects of the regular probiotic yoghurt consumption on reducing the numbers of cariogenic streptococci in the oral cavity [61,62,63,64] in both saliva and dental plaque. Further studies on periodontal diseases (gingivitis and periodontitis) presented to researchers the definite ability of certain probiotic lactobacilli to antagonize the pathogenic bacteria active in periodontitis such as *Porphyromonas gingivitis* and *Aggregatibacter* species [60,65]. The trend of these positive reports about the roe of probiotics in managing periodontal disease is of utmost significance if we couple this to the complexity of the etiology of periodontitis which is believed to be biofilm induced infection [66]. However, Bartold and Van Dyke [67] associated periodontal disease in general with imbalances with the host local microbiome pertaining to increased numbers of pathogens and reduced proportions of health associated bacteria [68]. Finally, since the aim of managing the oral cavity infections lies in reducing the pathogenic burden by antibiotics or other means, this effect is not a permanent process due to recolonization in due course [4], and there are problems associated with emerging resistant bacteria, considering probiotics and beneficial bacteria with their prospective disease preventive capabilities provides a reasonable option for safer oral health.

## 6. Halitosis and Probiotics

Halitosis (malodor) is primarily caused by anaerobic bacteria associated with periodontal diseases [69]. It has several causes stemming from imbalance of the oral cavity microbiota such as metabolic disorders, use of certain types of foods and some respiratory infections [64]. Cultivable oral cavity bacteria associated with halitosis include mainly *Porphyromonas gingivitis*, *Treponema denticola* and *Treponema forsythia* [70,71,72]. It is caused by the production of volatile sulfur compounds due to degradation of S-containing amino acids by these bacteria and others. The reduction of pathogenic bacterial counts involved in halitosis or its replacement through colonization with probiotic strains would elucidate treatment, management and control of halitosis [20,73,74]. Studies carried out on periodontitis and halitosis patients showed a high degree of heterogeneity of probiotic strains used, dosages, method and vehicle of administration, and treatment duration. Probiotic strain co-aggregating ability to dental pathogens may pave the way for wider application routes for reduction of halitosis symptoms through improving co-aggregation and/or colonization of selected probiotics to volatile sulfur compounds producing oral pathogens [4,74,75]. The complex oral microbiota is considered the major hurdle challenging researchers on prevention, treatment, management and control of dental diseases including halitosis. In future studies, it is necessary to obtain substantial data from blind and randomized large groups of patients with emphasis on strain efficacy, dose effects and most successful delivery vehicles.

## 7. Oral Fungal Infections

Interactions between bacteria and fungi in the oral cavity environment are dynamic and usually drive the structure and behavior of the oral cavity community resulting in pathogenesis of the oral diseases [76,77,78,79]. Vest et al. [76] studying the oral fungal communities reported diverse numbers of fungal genera including *Candida*, *Saccharomyces*, *Pencillium*, *Cladosporium*, *Malasseezia* and *Fusarium* with varying densities. However, species of *Candida* were dominant and they are known to be commensals in the oral cavity and present in about 25%–75% of the microbiota of healthy individuals [80]. These species are opportunistic pathogens and may under suitable conditions infect the oral mucosa causing infectious candidiasis [11]. The majority cases of candidiasis are associated with *Candida albican* isolates [77]. Other *Candida* species such as *C. krusei*, *C. tropicalis*, *C. glabrata*, *C. parapsilosis*, and *C. dubliniensis* were incriminated and isolated from oral cavity infections [78,79,80,81]. The oral cavity is among the most diverse microbiomes of the human body where different niches occur in the plaque, saliva and epithelial mucosa, thus eliciting dysbiosis by fungi and bacteria [82,83]. These dysbiotic infections could affect mucosal surfaces of the oral cavity and esophagus, and may become systemic [11]. Under certain stress conditions of debilitated patients, candidiasis would be life threatening and cause “diseases of the diseased” [84,85]. Considering the slow down in antifungals development [21] coupled with little number of new antibiotics available as well as the increased rate of emerging resistant fungal and bacterial strains [86,87], it has become very inviting to researchers and health professionals to extend the probable adoption of probiotics as an option in oral cavity care where probiotics may exert therapeutic or preventive effects on common oral diseases. Nevertheless, in vitro studies have several limitations and they never exactly mimic the microbiota of the oral cavity [11]. It is also recognized that probiotics activity is highly host and strain specific, even at strain level. Significant differences in growth inhibition and co-aggregation were observed, especially against *Candida* pathogenic species [11,85,88]. This reconfirms the notion that probiotics should not be put in one box and further in vitro and in vivo studies are needed to elucidate and understand the role of probiotics in prevention, treatment and management of fungal infections of the oral cavity. 

## 8. Designer Probiotics as a Base for Living Therapeutics

Recent studies on the mode of action of probiotics that target the oral cavity, though not fully substantiated by in vivo studies [89,90], are presented in several categories: competition for nutrients; growth factors; adhesion; production of inhibitory substances such as enzymes, antimicrobials, bacteriocins, and H_2_O_2_; inhibition of pathogen induced production of cytokines; and immune system stimulation [91]. All of these envisioned activities vary according to host and microbial strains [92,93]. In the last six years, interesting reviews about the probable and real use of probiotics have been published [88,89,90,92,93]. Although the feeling of low degree of substantiation in vitro studies prevailed, nevertheless most authors agreed upon the great promise of the benefits of probiotic use in oral health. They also pointed out that enhancing the functional repertoire of probiotic microorganisms as biotherapeutic agents is an attractive and promising approach. This approach is being pushed to the frontiers of research due to the slow down in new drugs development and increasing rate of emerging resistant pathogens. Probiotics able to deliver new and novel therapeutics are hopefully emerging with site specific and well-defined efficacy, which is now known as designer probiotics. These emerging living improved therapeutics [designer probiotics] will without doubt transform existing paradigms of disease prevention, control and management. These awaited designer probiotics would, when fully developed, expand the efficiency and efficacy of probiotics by introducing new genetic circuits to develop new drug delivery systems [94,95,96]. As a result of pressing needs for alternative biotherapeutics and nutraceuticals, the science of probiotics has emerged in the post genomic era of medicine and biology as a hot research area in the quest for better healthcare areas including the oral cavity. In this context, the use of designer probiotics is being tested against infectious diseases and in anticancer therapy studies [97,98]. When used as dietary supplements or applied topically, designer probiotics would support normal physiology and immunity to improve health and prevent infections, oxidation stress, autoimmune responses and inflammatory diseases [99]. As for dental and periodontal health, an array of probiotics has demonstrated beneficial effects. Clinical studies have typically used surrogate endpoints such as *Streptococcus mutans* counts, salivary flow, plaque or gingival scores, and pocket depth to substantiate efficacy [92,93,94]. These studies provided promising outlook, however, need to be further confirmed in randomized double blind placebo studies with specific target sites in the oral cavity.

## 9. Concluding Remarks and Future Directions

In medical settings, it is now recognized that the increase in emerging resistant pathogens coupled to metabolic diseases are paramount public health and oral health concerns. This concern necessities the search for safe, cost-effective and inventive alternatives and/or complimentary means to the traditional uses of prophylactics and treatment. In this context, probiotics, both general and designer, would offer potential prospects. This conviction of the importance of biotherapeutics that integrate with clinical prescriptions should be inviting and given priority in medical research. The potential role of such living biotherapeutics should not overlook the notion that probiotic-mediated antagonism and functional characteristics may hinder some commensal bacteria and/or inter signals from indigenous microbiota. The effect of this prospective hindrance would be reduced through designer probiotic development, which needs concerted research efforts to fully understand and substantiate their safe use, specificity and efficacy. This becomes imminent if we know that most of the documented health benefits come from research and clinical trials on animal models. With all of this in mind, it appears that the stage is set not only for probiotics use in medicine but also expansion to include the oral cavity microbiome. A further outlook might be to have the personal oral microbiome characterized to the genus level by providing a saliva sample to be used as a biomarker to formulate a list of probiotic strains specifically targeting resident pathogenic bacteria. This, no doubt, will help in offering a strong complementary and/or alternative approach for the next generation [designer] probiotics for oral cavity and throat healthcare.

## Figures and Tables

**Table 1:** Totally or partially proven probiotic activity and mode of action on oral pathogens.

Probiotic	Activity	References
*S. salivarius* K12	Antagonism	[10]
*L. reuteri*	Coaggregation	[11]
*S. salivarius* K12, M18	Interaction withepithelium	[12]
*L. acidophilus* LA-5	Modulation of biofilm	[13]
*L. casei* LC-11	Reduction of cariogenic biofilm potential	[13]
*L. paracasei*	Caries management	[14,15]
*Lactobacilli* sp	Periodontal control	[16]
*Bifidobacterium* sp	Gingivitis management	[16]
*L. rhamnosus* GG	Modulation of immune response	[17]
*Bifidobacterium*	Improved resistance	[16,18]
*Animalis subsp. lacis*	to oral infections	
